# Supplementary material for: Turning toward or away from God: COVID-19 and changes in religious devotion
Source: PLoS One. 2023 Mar 8;18(3):e0280775. doi: 10.1371/journal.pone.0280775 (PMC9994730; doi:10.1371/journal.pone.0280775)
Supplement: S1 Table — Factors are indicated by bold values. (DOCX) [file pone.0280775.s001.docx]

Table S1

*Exploratory factor analysis for COVID-19 belief items*

| COVID-19 Belief Items | Factor 1 | Factor 2 | Factor 3 | Factor 4 |
| --- | --- | --- | --- | --- |
| **Regulations** |  |  |  |  |
| I believe it is time to end the sheltering at home | -.20 | -.04 | .01 | **.76** |
| I believe it is time to get the economy back up and running | -.09 | -.00 | .05 | **.85** |
| **Health** |  |  |  |  |
| How serious of a threat do you believe COVID-19 is to your personal health? | **.74** | .16 | -.10 | .06 |
| How serious of a threat do you believe COVID-19 is to the health of your family members? | **.78** | .07 | -.05 | .03 |
| How serious of a threat do you believe COVID-19 is to the health of your country? | **1.02** | -.09 | .05 | .07 |
| How serious of a threat do you believe COVID-19 is to world health? | **.96** | -.12 | .11 | .03 |
| **Personal Finance** |  |  |  |  |
| How serious of a threat do you believe COVID-19 is to your financial situation? | -.05 | **.89** | .03 | .04 |
| How serious of a threat do you believe COVID-19 is to your family’s financial situation? | -.04 | **.92** | .05 | -.01 |
| **World Finance** |  |  |  |  |
| How serious of a threat do you believe COVID-19 is to your country’s economy? | .07 | .08 | **.83** | .02 |
| How serious of a threat do you believe COVID-19 is to the world economy? | -.03 | -.01 | **1.00** | -.07 |
| **Items Evaluated Individually due to Poor Loading**  It is morally wrong to violate social distancing policies right now  How much stress do you feel from COVID-19  I believe that God will protect me from COVID-19 | .44  .32  .21 | .02  .44  .13 | .03  -.02  -.12 | -.18  .07  .35 |

Factors are indicated by bolded values.
