# Supplementary material for: Turning toward or away from God: COVID-19 and changes in religious devotion
Source: PLoS One. 2023 Mar 8;18(3):e0280775. doi: 10.1371/journal.pone.0280775 (PMC9994730; doi:10.1371/journal.pone.0280775)
Supplement: S2 Table — Factors are indicated by bold values. (DOCX) [file pone.0280775.s002.docx]

Table S2

*Exploratory factor analysis for COVID-19 social distance motivation items*

| Social Distance Motivation | Factor 1 | Factor 2 |
| --- | --- | --- |
| **Avoid Sickness** |  |  |
| To avoid getting sick  To avoid getting others sick  To protect others who are vulnerable  To help the global community  To avoid getting my family sick  **Avoid Punishment**  Because the government has required it  So I don’t get in trouble | .66  .86  .80  .68  .70  .08  -.04 | .03  -.08  -.06  .09  .06  .61  .77 |

Factors are indicated by bolded values.
