# Supplementary figures and images for: Turning toward or away from God: COVID-19 and changes in religious devotion
Source: PLoS One. 2023 Mar 8;18(3):e0280775. doi: 10.1371/journal.pone.0280775 (PMC9994730; doi:10.1371/journal.pone.0280775)

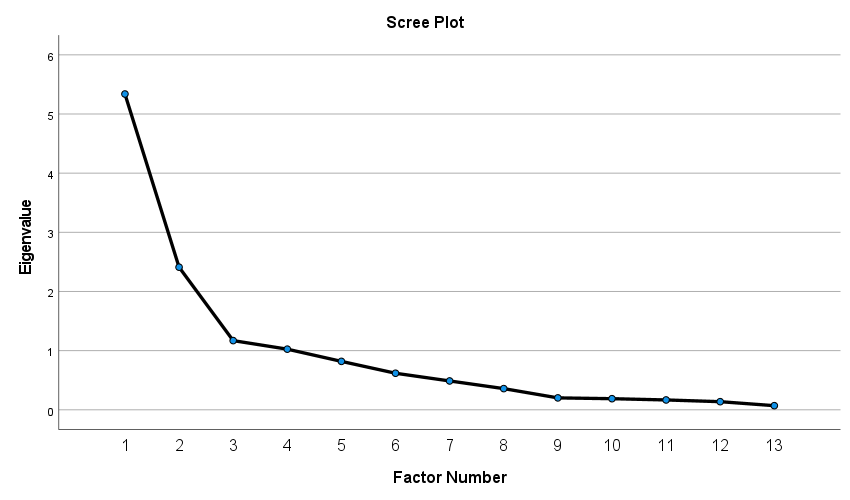


*Figure S1*. Eigenvalue scree plot for COVID-19 belief items.

Supplement: S1 Fig — (DOCX) [file pone.0280775.s003.docx]

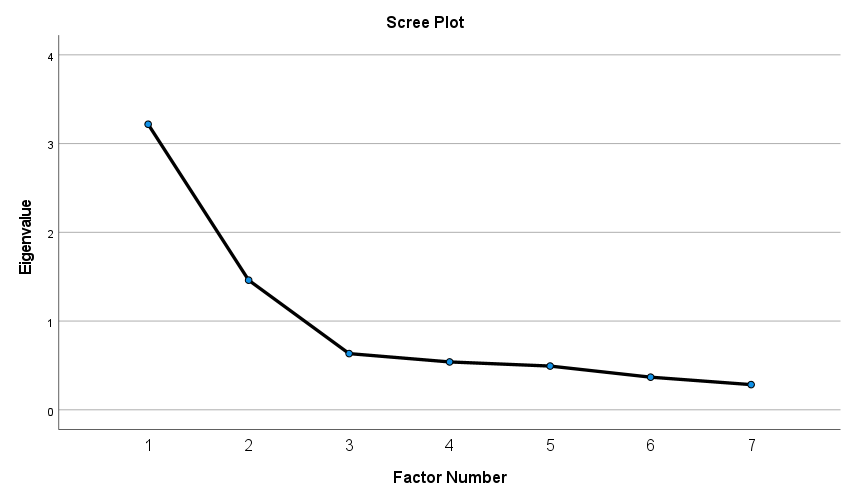


*Figure S2*. Eigenvalue scree plot for COVID-19 social distancing motivation items.

Supplement: S2 Fig — (DOCX) [file pone.0280775.s004.docx]
