# Supplementary material for: Turning toward or away from God: COVID-19 and changes in religious devotion
Source: PLoS One. 2023 Mar 8;18(3):e0280775. doi: 10.1371/journal.pone.0280775 (PMC9994730; doi:10.1371/journal.pone.0280775)
Supplement: Attachment — Submitted filename: Response to Reviewers.docx [file pone.0280775.s006.docx]

Andrea Fronzetti Colladon, Ph.D.
Academic Editor
PLOS ONE

Dr. Colladon,

We appreciate the thoughtful comments throughout this revision process. We believe we have been able to address the last comments from Reviewer 2 for this “Minor Revision” decision. If there is anything else that we can do to clarify points or address concerns, we are happy to do so.

**Reviewer 2’s Comments**

**Comment: I would like to thank the authors for considering my suggestions and for updating the manuscript. The revised version introduced some improvements and is now more transparent about the entire research process. This is an important change because such transparency will allow readers to judge what can be inferred from this study (given the relatively large number of variables tested).**

**Response:** We appreciate the compliment!

**Comment: After reading the revised version I stand with my previous assessment of this study – the findings are relatively trivial (showing variables correlating with increase and/or decrease in religiosity), and the most interesting question posed by the authors remains unanswered: that is, why some people react to stress by an increase in religiosity, some do nothing, and some with decrease? The importance of this question is illustrated by the positive association of an increase in religiosity with variables indexing well-being and prosocial emotions. Hence, not properly addressing this question feels a bit frustrating for me as a reader. To be fair, the authors provide a hypothetical answer to this question relying on internal and external religiosity (p. 34). However, this answer is not supported by the presented data because these variables were not assessed.**

**Response:** We share this reviewer’s frustrations regarding the limitations of the data collected in this study. Particularly with cross-sectional data there are challenges to be fully confident in some of the potential explanations we suggest. We have done our best to acknowledge this in the limitations section. We do, however, think that the strength of combining quantitative and qualitative data help us to make some tentative assertions. We appreciate the reviewer raising the point specifically about internal versus external religiosity as it helped us realize that we can more clearly point to the qualitative evidence in support of our assertions. On pg. 33-34, we have added the following statement to more clearly highlight some additional evidence on this point of internal and external religiosity:

“Our qualitative data, in particular, seem to speak to this distinction between focusing on more internal versus external religiosity. Among those who reported an increase in religious devotion, 47.7% participants focused their comments specifically on engaging in personal worship such as prayer or scripture study; alternatively, among those who reported a decrease in religious devotion, 68.9% mentioned the lack of engagement in communal worship. This difference is striking given that we did not specifically prompt participants to report on these experiences denoting internal or external religiosity. Rather they spontaneously reported them in response to a broad, open-ended question about “Why” their religious devotion had changed or not changed since COVID-19.”

While we acknowledge our inability to state with complete confidence what factors drive the difference between the increase in religious devotion and decrease in religious devotion groups, we hope that this reminder of additional qualitative evidence at least adds to the credibility of our assertions about this internal versus external focus being part of the picture.

**Comment: From the sheer number of results the authors provide, a different (speculative) explanation seems probable. Namely, that people who were already susceptible to a decline in the intensity of their faith did so due to Covid-related stress and demands. Looking at the first block of results in Table 1 (Sociodemographic), no statistically significant differences were reported. However, if we look at the overall profile of people who decreased in their religiosity, we see that they have, on average, higher education, fewer kids, higher income, belong to a higher social class, and are liberal. In one way or another, all these variables have been implied in the increasing secularization of Western countries. Why these variables were not significant may have to do with the chosen statistical test…**

**I would be personally happy to see just a model where the probability of belonging either to a decrease or increase category would be predicted by an interaction between Covid-related stress and some of the sociodemographic variables (education, social class, political affiliation). However, I concur with Reviewer #1 that a path model would be more revealing.**

**Response:** This is an intriguing theory. As requested, we conducted analyses with Covid-related stress interacting with education, social class, and political affiliation, predicting the likelihood of increasing versus decreasing in religious devotion (dichotomized, as we believe this best fits the description of what the reviewer is suggesting). We conducted the analyses with all predictors simultaneously, as well as separate models of each predictor interacting with Covid-related stress. All analyses were conducted with path models, consistent with the suggestion of the reviewer. We did not find any significant results. If there are further analyses on this point that are desired, we are open to them. We have posted the analyses on OSF.

Just because we did not find anything does not negate the value in this point. We still believe this is worth exploring more deeply in other data. We have added information to the discussion section (pg. 37-38) highlighting that it would be valuable to assess this more closely in future research.

“We note that although there were not significant differences between groups for sociodemographic variables, that the means for those in the decrease group seemed to suggest higher education, fewer kids, higher income, belonging to a higher social class, and being liberal. As these variables are connected to secularization of Western countries, it could be valuable for future research to more closely explore whether those who are already susceptible to a decline in the intensity of their faith may have done so due to COVID-19 related stress and demands.”

**Comment: In response to my argument that the data should be analyzed using regressions, the authors made several statements that do not seem entirely correct to me. Let me explain point by point.
The authors wrote: “Our first concern was in potentially having too much confidence in directional propositions. With MANOVA analyses, we have been careful to try and use language surrounding group differences when discussing our analyses, rather than language surrounding prediction, as our analyses cannot determine the direction of association.” I agree, but the same applies to regression coefficients, which are identical to MANOVA if the regression equation is set up with the same parameters as MANOVA! A simple regression coefficient is nothing else than a correlational coefficient. Thus, adjusting these estimates for variables that could potentially confound the assumed causal link is even more important.**

**Further, the authors argued: “we are concerned that some results may become more challenging to interpret if we are forced to make change in religious devotion exclusively an outcome, or exclusively a predictor.” Again, the regression equation is set as if some variables are predictors and some outcomes, but it’s still the same result even if those variables are switched (and the same will be true for MANOVA!). Hence, this is not about the test being used but about the assumptions that should be explicitly stated.**

**Next, the authors wrote: “were concerned about the multicollinearity of so many correlated predictors simultaneously predicting group membership. Particularly with reinserting variables from the supplement, this would add up to 37 simultaneous predictors.” Good point, yet it begs the question why the authors measured 37 predictors? …**

**Next point: “Alternatively, perhaps the three groups could be modeled as several dichotomized categories through dummy variables. But this may still create some problems as this would involve multiple dummy variables potentially being highly correlated enough to create some multicollinearity concerns.” Again, this is exactly what MANOVA does. Creating dummy variables based on the change in religiosity will produce the same results in linear regression as in MANOVA.
Finally, “these analyses are preregistered and we were concerned about deviating too heavily from analyses we specifically committed to conduct.” This is a valid point. In my view, however, the point of pre-registration is to be transparent about the research process. What is better – rigidly sticking to an inferior analytical approach or explaining the change to a more informative approach?
Ultimately, the decision about which statistical test to use is up to the authors. All have their pros and cons. In my replies, I just wanted to point out that the MANOVA results can be reproduced using linear regression and that the regression approach offers further flexibility to make the models more informative.**

**Response:** The reviewer has brought up several fair points. We believe that a regression approach could be justified. We appreciate, however, the acknowledgment that the statistical test is ultimately up to us as authors. With this acknowledgment, we maintain that the MANOVA approach is still defensible and best suits the purposes of this particular paper.

A primary concern with switching to a regression approach concerns the fact that the language of prediction inherent in the use of this approach often insinuates directionality, which of course we cannot determine from our cross-sectional data. There is room for disagreement here, as the reviewer correctly makes a fair point about regressions really not being more than correlation coefficients. But we believe it would be more challenging to navigate this particular manuscript with language of prediction than language of group differences from MANOVA. Additionally, we believe the results become more digestible and interpretable with such clear cut groups and group difference language, especially since they are they same groups used for the qualitative coding.

Another concern with a potential regression approach is that we do have 37 variables to assess; we appreciate the reviewer’s agreement that this many variables in a regression would likely create problems with multicollinearity. It is true that 37 variables is a lot. In writing the manuscript, we began to realize how things could potentially become unwieldy (which is why in our original submission we placed many of the results in a supplemental file). We did, however, accept the feedback of the reviewer and editor in reinserting all of the variables that were initially preregistered. Though it is a lot of variables, we did our best to ensure there were at least theoretically grounded reasons for why these variables may be valuable to include. We maintain, consistent with our preregistration that all the variables have at least some theoretical basis for how they might explain group differences between those who increase, stay the same, or decrease in religiosity. With the exploratory nature of the study it made sense to account for them. With all of them needing accounting, we maintain that a MANOVA is beneficial in avoiding potential multicollinearity concerns that would likely emerge from a regression approach.

Finally, at the end of the day, we ultimately did preregister MANOVA analyses. An argument could be made for alternative analyses. Considering, however, there is not necessarily anything incorrect in the use of MANOVA, and the acknowledgment from the reviewer that the decision is up to us as authors, we prefer to stay consistent with what we preregistered.

**Comment: Thank you for sharing your questionnaire. It did not help me to understand the data set, though. The order of variables does not seem to follow the order of questions, and I was still confused about what variable indexes which question. A proper codebook explaining each variable and its range would be necessary.**

**Response:** We apologize for the lack of clarity for some aspects of the codebook. We have ensured that all variables are presented in the same order in the codebook and dataset. We also have added all the stems in the codebook that are used to label variables in the dataset (e.g., the stem of “awe” being in parentheses next to the awe measure in the codebook so that interested readers can more clearly see that the six items for awe in the dataset have the stem of “awe”, such as awe1, awe2, etc.). We also have made sure that the range is shown in the codebook for all quantitative measures.
